# Traumatic Experiences, Psychological Distress and Suicide‐Related Behaviors in Autistic Adults

**DOI:** 10.1002/aur.70137

**Published:** 2025-11-25

**Authors:** T. A. Chikaura, E. Weir, S. Griffiths, T. Procyshyn, M. Pelton, C. Allison, H. Hodges, S. R. White, T. Ford, S. Baron‐Cohen

**Affiliations:** ^1^ Autism Research Centre, Department of Psychiatry University of Cambridge Cambridge UK; ^2^ Child and Adolescent Resilience and Mental Health, Department of Psychiatry University of Cambridge Cambridge UK; ^3^ Department of Clinical, Educational and Health Psychology, Division of Psychology and Language Sciences University College London London UK

**Keywords:** autism, mental health, self‐harm, substance use, suicide attempts, suicide plans, trauma

## Abstract

Autistic adults have increased risks of trauma, suicide, and poor mental health compared to non‐autistic adults, with 1 in 4 autistic adults attempting suicide. We administered an anonymized, self‐report survey to 424 autistic and 345 non‐autistic adults through a convenience sampling framework. Binomial logistic regression models identified whether trauma and autism diagnosis were related to (i) self‐harm, (ii) suicide attempts, (iii) suicide plans, (iv) a mental health condition that impacts daily life, and (v) substance use to cope. Heatmaps were generated to identify traumas that frequently co‐occur with psychological distress and SRB. After accounting for trauma and demographic differences, autism remained a significant predictor of all outcomes, except whether individuals used substances to cope (OR: 0.78, 95% CI: 0.54–1.12, *p* = 0.18). Autistic people were more likely to report self‐harm (OR: 2.71, 95% CI: 1.85–4.00, *p* < 0.01), suicide attempts (OR: 2.45, 95% CI: 1.65–3.68, *p* < 0.01), suicide plans (OR: 2.00, 95% CI: 1.41–2.83, *p* < 0.01), and experiencing a mental health condition that impacts daily life (OR: 3.58, 95% CI: 2.42–5.33, *p* < 0.01) than non‐autistic people. Among autistic people, childhood victimization co‐occurred with a mental health condition that impacts daily life, self‐harm, and suicide plans most frequently. This study provides evidence of complex relationships between autism, trauma, self‐harm, suicide attempts, suicide plans, and a mental health condition that impacts daily life. Focusing on the prevention of trauma, coping strategies, and recovery from traumatic events through safeguarding and support may be critical tools for suicide prevention among autistic people.

## Summary

This study explored whether several lifetime traumas and having an autism diagnosis were associated with self‐harm, suicide attempts, suicide plans, a mental health condition that affects daily life, and regularly using alcohol or another (non‐prescribed) drug in order to cope. We found that, even after accounting for trauma, autistic people were still more likely to report self‐harm, suicide attempts, suicide plans, and a mental health condition impacting daily life compared to non‐autistic people; in addition, different traumas were associated with different types of psychological distress and suicide‐related behaviors (SRB). These findings can inform international public health policy and clinical care to provide tailor‐made mental health support after trauma.

**Abbreviations**
SRB: suicide‐related behaviors
VEQ: vulnerability experiences quotient

## 1 Introduction

Growing evidence suggests that negative mental health outcomes such as depression, anxiety, substance use, self‐harm, and suicidality are associated with traumatic experiences in autistic people (Taylor and Gotham 2016; Griffiths et al. 2019; Weir et al. 2021; Mandell et al. 2005; Pelton et al. 2020; Hoover and Kaufman 2018). Despite increasing efforts to destigmatize autism and establish autistic‐led communities and advocacy groups (Jordan 2010; Jaarsma and Welin 2012), autistic people can still be perceived negatively (Botha et al. 2022) and may feel the need to mask their autistic traits in order to fit in with societal expectations (Allely 2018; Livingston et al. 2019; Cage and Troxell‐Whitman 2019). A meta‐analysis found that the pooled prevalence for trauma in autistic people was 44% (Trundle et al. 2023). Additional subgroup analysis found a pooled prevalence of 84% in autistic people who have experienced various types of traumas such as bullying, child abuse and crime victimization (Trundle et al. 2023).

Autistic people are far more likely to have co‐occurring mental health (Croen et al. 2015; Lever and Geurts 2016) and neurodevelopmental conditions (Leyfer et al. 2006) compared to non‐autistic people. Approximately 54% to 79% of autistic adults receive at least one psychiatric diagnosis (Croen et al. 2015; Lever and Geurts 2016) with anxiety and depression being the most common (Lever and Geurts 2016; Martini et al. 2022a). The current and lifetime prevalence estimates of attention‐deficit hyperactivity disorder (ADHD) among autistic people are 38.5% and 40.2%, respectively (Rong et al. 2021). These co‐occurring conditions are often clinically unrecognized or misdiagnosed, and increase the risk of long‐term negative outcomes for autistic people (Gillberg et al. 2016; Sikora et al. 2012; Stewart et al. 2006). For example, autistic children with clinically significant ADHD characteristics are at an increased likelihood of difficulties in adaptive functioning and poorer health outcomes compared to autistic children with fewer ADHD characteristics (Sikora et al. 2012). Similarly, depression has been linked with self‐injury in autistic people (Stewart et al. 2006).

Autistic people are at increased risk of premature mortality, with suicide as a key contributor, compared to non‐autistic people (Hirvikoski et al. 2016). A systematic review based on international data highlights that 1 in 4 autistic adults attempts suicide and 1 in 3 has suicidal ideation (Brown et al. 2024). A study from the United States of America found that autistic females are more than three times more likely to die by suicide than non‐autistic females (Kirby et al. 2024). In Taiwan, autistic individuals between the ages of 12 and 29 are nearly six times more likely to attempt suicide, and autistic people attempt suicide at a significantly younger age, compared to non‐autistic people (Chen et al. 2017). What is clear is that, globally, autistic people are at a higher risk of experiencing self‐harm, suicidal thoughts, suicide plans, suicide attempts, and death by suicide compared to their non‐autistic peers (Hirvikoski et al. 2016; Brown et al. 2024; Kirby et al. 2024; Chen et al. 2017). Suicide prevention among autistic people remains a notable challenge because the strategies applied to the general population often fail to meet the needs of autistic people (Cassidy and Rodgers 2017).

Suicide is difficult to study, and its complex pathways make it difficult to design interventions. Existing theories suggest that the mechanisms underlying suicidal thoughts are different from those that govern suicide attempts, suggesting that suicidality should not be studied as a single construct (Joiner 2005; O'connor 2011; Klonsky et al. 2021). Additionally, these theories recognize trauma as a key factor in the development of suicidality (Joiner 2005; O'connor 2011; Klonsky et al. 2021). Although we do not know how trauma presents itself in autistic people, existing literature suggests that autistic and non‐autistic people experience trauma differently. Kerns and colleagues propose a transactional relationship between trauma and autism. In this hypothetical framework there are unique aspects of autism that may influence how trauma is (i) encountered, (ii) perceived, (iii) experienced, (iv) related to the development of negative mental health outcomes, and (v) presented in behaviors and/or outcomes. Similarly, trauma may directly exacerbate the presentation of autism‐related traits, such as sensory sensitivities, or indirectly through deleterious outcomes such as suicidality (Kerns et al. 2015).

While several studies have examined suicide risk factors among autistic people (Pelton et al. 2020; Martini et al. 2022b; Cassidy et al. 2014; Cassidy et al. 2018; Kõlves et al. 2021; McDonnell et al. 2020; Lai et al. 2023; Hirvikoski et al. 2020; Hand et al. 2020; van Bentum et al. 2024; Licence et al. 2020; Costa et al. 2020; Hwang et al. 2019; Pelton and Cassidy 2017; Arwert and Sizoo 2020; Cassidy et al. 2020; Hosozawa et al. 2021; Raymaker et al. 2020), only one study has explored lifetime trauma and lifetime suicidality in autistic people (Pelton et al. 2020). This study showed that trauma has an attenuated effect on lifetime suicidality in autistic people (Pelton et al. 2020). However, the same study presented suicidality as a single construct and did not explore the relationship between several lifetime traumas and different types of lifetime psychological distress and suicide‐related behaviors (SRB). At present, we do not understand the associations between trauma and specific SRB, such as how trauma relates to suicide plans compared with its relationship to suicide attempts. Throughout the paper we will use the terms psychological distress and SRB to refer to the following outcomes collectively: *self‐harm, suicide plans, suicide attempts, substance use as a coping strategy, and mental health conditions that impact daily life*.

Given that autistic people are at a greater risk of exposure to lifetime trauma compared to non‐autistic people (Taylor and Gotham 2016; Griffiths et al. 2019; Trundle et al. 2023; Moseley et al. 2021), there is an urgent need to identify how different types of lifetime trauma relate to lifetime psychological distress and SRB. Using secondary data analysis of data from an existing, anonymized, self‐report survey (Griffiths et al. 2019), this study aimed to address this lack of evidence. This is distinct from the previous work because we analyzed the relationship between the number of traumatic experiences with psychological distress and SRB, rather than the frequency of traumatic experiences on its own. We identified, for the first time, the different types of lifetime traumas that frequently co‐occur with lifetime psychological distress and SRB in autistic adults. Specifically, this study investigated whether:

- Higher levels of trauma are associated with psychological distress and SRB in autistic people and non‐autistic people.
- Autism diagnosis has an impact on psychological distress and SRB after accounting for trauma.
- Different types of traumas are associated with different types of psychological distress and SRB in autistic and non‐autistic people.

## 2 Methods

### 2.1 Participants and Recruitment

Participants were recruited through the Cambridge Autism Research Database [CARD] [www.autismresearchcentre.com], the Cambridge Psychology participant database [www.cambridgepsychology.com], charity websites such as Autistica [e.g., www.autistica.org.uk], and on social media channels such as X. Participants were informed that the online survey was about autism, vulnerability, and mental health. Nine hundred and forty participants completed the survey; 168 people were excluded because of missing data for country of residence, sex, age, education level, predictor, or outcome variables. Another three people were excluded who selected ‘other’ for sex, as a result of perfect separation issues due to small sample size. This resulted in a final sample of 769 individuals, including 424 autistic adults. A priori power calculations confirmed that this sample size was sufficient to conduct well‐powered analyses. Participants in the autism group self‐reported their diagnosis from a recognized, qualified clinician (psychiatrist, clinical psychologist, neurologist, pediatrician). Non‐autistic participants confirmed that they did not have an autism diagnosis or suspect they might be autistic.

The sample predominantly comprised of UK residents, people assigned female at birth, and individuals who completed at least one tertiary educational qualification. There were significant group differences in mean age, sex assigned at birth, gender identification, country of residence, and average autism spectrum quotient (AQ‐10) scores between autistic and non‐autistic groups. Full results are available in Table 1.

**TABLE 1:** Participant characteristics.

Characteristics	Autistic (424)	Non‐autistic (345)	*p*
Mean age in years (SD)	43.60 (14.18)	49.82 (14.91)	**< 0.01**
Sex assigned at birth, *N* (%)			**< 0.01**
Female	246 (58.02)	247 (71.59)	
Male	178 (41.98)	98 (28.41)	
Gender Identification, N (%)			**< 0.01**
Cisfemale	205 (48.35)	242 (70.15)	
Cismale	185 (43.63)	97 (28.12)	
Non‐binary/other	34 (8.02)	6 (1.74)	
Education, *N* (%)			0.11
Further vocational qualifications	85 (20.05)	55 (15.94)	
High school^a^	78 (18.40)	53 (15.36)	
University undergraduate level qualification	119 (28.07)	94 (27.25)	
University post‐graduate level qualification	142 (33.49)	143 (41.45)	
Country of residence, *N* (%)			**0.02**
United Kingdom	298 (70.28)	216 (62.61)	
United States of America	57 (13.44)	73 (21.16)	
Other	69 (16.27)	56 (16.23)	
Trauma, mean (SD)^b^	19.44 (8.27)	12.52 (7.33)	**< 0.01**
Female, mean (SD)	20.3 (7.83)	13 (7.72)	
Male, mean (SD)	18.3 (8.74)	11.3 (6.12)	
AQ‐10 score, mean (SD)^c^	7.86 (1.96)	3.50 (2.73)	**< 0.01**
Female, mean (SD)	8.07 (1.86)	3.21 (2.67)	
Male, mean (SD)	7.57 (2.06)	4.23 (2.76)	

*Note:* Values in bold indicate *p* values less than 0.05.

^a^ High school variable includes people that answered whether they had a high school qualification or no formal qualification. Both variables were collapsed due to low numbers in each independent variable.

^b^ Trauma *p* value is for the group difference between autistic and non‐autistic people.

^c^ AQ‐10 score *p* value is for the group difference between autistic and non‐autistic people.

### 2.2 Measures

Traumatic experiences were measured by the Vulnerability Experiences Quotient (VEQ) (Griffiths et al. 2019). This scale illustrated good internal reliability for the autistic (Cronbach's *α* = 0.89) and non‐autistic (Cronbach's *α* = 0.88) groups (Griffiths et al. 2019). More information on the VEQ is provided in the Supporting Information. We employed a categorical measure of trauma (0–5, 6–10, 11–15, 16–53 traumas), as there wasn't a linear relationship between the number of traumatic experiences and the risk of psychological distress and SRB, as confirmed by the residuals in the quantile‐quantile plots. Psychological distress and SRB were assessed with the VEQ mental health difficulties subdomain (see Supporting Information for additional information).

Autistic traits were measured by the short version of the autism spectrum quotient (AQ‐10) (Allison et al. 2012). The AQ‐10 has shown good sensitivity (0.88) and specificity (0.91) for detecting autism in a diagnosed sample at a cut‐point of six or above in other studies (Allison et al. 2012). In this study 57.6% of our participants met the criteria for high autistic traits. Further details on the AQ‐10 can be found in the Supporting Information.

Covariates were coded in the following ways: age (continuous), sex assigned at birth (binary), country of residence (categorical: United Kingdom (UK), United States of America (USA), and Other), education (categorical: high school qualification, further vocational qualifications, undergraduate‐level qualification, and postgraduate‐level qualification), and 2+ neurodevelopmental/mental health conditions (binary: 0/1 vs. 2 or more of the following: anxiety disorder, language delay, attentional deficit hyperactivity disorder (ADHD)/attentional deficit disorder (ADD), obsessive compulsive disorder (OCD), oppositional defiant disorder (ODD), bipolar disorder, panic disorder, conduct disorder, personality disorder, depression, post‐traumatic stress disorder, dyslexia, schizophrenia/psychosis, dyspraxia/developmental coordination disorder, sensory processing disorder, eating disorder, social phobia/social anxiety disorder, intellectual disability, specific phobia, generalized anxiety disorder, and Tourette syndrome/tic disorder).

### 2.3 Ethics Statement

The Psychology Research Ethics Committee, University of Cambridge (PRE.2017.031) approved the original survey study. The Ethics Committee confirmed that further ethical approval was not required for this secondary data analysis.

### 2.4 Design and Statistical Analysis

All statistical analyses were performed using RStudio 4.2.2. Descriptive statistics were computed to summarize and describe the characteristics of the study variables via base R functions, including Chi‐square, Mann–Whitney U, and independent t‐tests.

Four binomial logistic regression models adjusting for increasingly more covariates were generated using the *glm function* within the *stats* package of R. Model 1 was unadjusted, Model 2 included autism as well as demographics, Model 3 added traumatic experiences, and Model 4 added 2+ co‐occurring mental health and/or neurodevelopmental conditions. A multiple comparison correction, with a *p* value threshold of 0.05, was applied to reduce Type I errors using the Benjamini–Hochberg methods (Benjamini and Hochberg 1995).

Two heatmaps were generated to visualize the co‐occurrence between lifetime trauma and lifetime psychological distress and SRB among autistic and non‐autistic people using *ggplot2*. We were interested in identifying the proportion of people who experienced each specific type of trauma alongside each specific type of psychological distress and SRB.

We ran supplementary analyses to consider how the level of autistic traits (rather than autism diagnosis) relates to trauma, psychological distress, and SRB. The results were largely similar to the main analyses and have been reported in the Supporting Information.

### 2.5 Community Engagement

The VEQ survey was co‐created by researchers, clinicians with experience of working with autistic adults in the UK National Health Service, and an advisory board with eight autistic adults (Griffiths et al. 2019). Initially, the researchers reviewed literature on lifetime traumas and the advisory board helped in deciding the traumatic experiences that were particularly relevant for the autism community. The advisory board, alongside clinicians, gave feedback on the language used in the survey. This participatory approach was essential in ensuring that the autistic community's needs were at the center of the research (Nicolaidis et al. 2011). Upon obtaining study results, we hosted a focus group discussion and presented our findings to nine autistic adults with lived experience of trauma and severe mental health distress. The lived experience experts were recruited through the Cambridge Autism Research Database (CARD) and the majority were white and identified as men. The mean age was approximately 48 years. The focus group members shared their personal experiences with severe mental health distress. Focus group members also helped with the interpretation and understanding of key themes in our findings, such as the way substances are used to cope by autistic adults.

## 3 Results

### 3.1 Autism and Trauma Are Associated With Psychological Distress and Suicide‐Related Behaviors

After accounting for trauma and demographic differences, autistic people were more likely to report self‐harm (OR: 2.71, 95% CI: 1.85, 4.00, *p* value: < 0.01), suicide attempts (OR: 2.45, 95% CI: 1.65, 3.68, *p* value: < 0.01), suicide plans (OR: 2.00, 95% CI: 1.41, 2.83, *p* value: < 0.01), and a mental health condition that affects their day‐to‐day lives (OR: 3.58, 95% CI: 2.42, 5.33, *p* value: < 0.01) than non‐autistic people. Autistic people remained at an increased likelihood of reporting self‐harm (OR: 2.36, 95% CI: 1.59, 3.52, *p* value: < 0.01), suicide attempts (OR: 2.08, 95% CI: 1.38, 3.16, *p* value: < 0.01), and suicide plans (OR: 1.68, 95% CI: 1.17, 2.40, *p* value: 0.01) after accounting for trauma and multiple co‐occurring mental health and/or neurodevelopmental conditions. Irrespective of trauma and multiple co‐occurring conditions, autism diagnosis was not associated with the use of substances such as alcohol to cope. Results on autism, lifetime trauma, psychological distress, SRB, and co‐occurring mental health and/or neurodevelopmental conditions are reported in Table 2. Full results can be found in Table S1.

**TABLE 2:** Logistic regression exploring the association between traumatic experiences and self‐harm, suicide attempts, suicide plans, a daily mental health condition, and substance use to cope in autistic and non‐autistic adults.

	Model 1^a^		Model 2^b^		Model 3^c^		Model 4^d^	
	Odds ratio (95% CI)	*p*	Odds ratio (95% CI)	*p*	Odds ratio (95% CI)	*p*	Odds ratio (95% CI)	*p*
Self‐harm								
Autism diagnosis	4.90 (3.59, 6.73)	< **0.01**	4.83 (3.43, 6.87)	< **0.01**	2.71 (1.85, 4.00)	< **0.01**	2.36 (1.59, 3.52)	< **0.01**
Trauma (6‐10)	—	—	—	—	1.87 (0.64, 6.83)	0.29	1.65 (0.57, 6.06)	0.40
Trauma (11‐15)	—	—	—	—	4.56 (1.66, 16.14)	**0.01**	3.56 (1.28, 12.70)	**0.03**
Trauma (16‐53)	—	—	—	—	16.80 (6.28, 58.75)	< **0.01**	11.06 (4.04, 39.15)	< **0.01**
2+ MH/ND conditions^e^	—	—	—	—	—	—	2.40 (1.63, 3.53)	< **0.01**
Suicide attempts								
Autism diagnosis	4.29 (3.02, 6.19)	< **0.01**	4.23 (2.93, 6.21)	< **0.01**	2.45 (1.65, 3.68)	< **0.01**	2.08 (1.38, 3.16)	< **0.01**
Trauma (6‐10)	—	—	—	—	5.80 (1.13, 106.24)	0.10	4.82 (0.93, 88.70)	0.14
Trauma (11‐15)	—	—	—	—	8.53 (1.73, 154.65)	0.05	6.13 (1.22, 111.67)	0.09
Trauma (16‐53)	—	—	—	—	31.99 (6.74, 573.18)	< **0.01**	18.93 (3.89, 341.51)	**0.01**
2+ MH/ND conditions^e^	—	—	—	—	—	—	3.01 (2.01, 4.56)	< **0.01**
Suicide plans								
Autism diagnosis	3.52 (2.61, 4.78)	< **0.01**	3.34 (2.44, 4.59)	< **0.01**	2.00 (1.41, 2.83)	< **0.01**	1.68 (1.17, 2.40)	**0.01**
Trauma (6‐10)	—	—	—	—	2.92 (1.16, 8.93)	**0.04**	2.54 (1.00, 7.81)	0.08
Trauma (11‐15)	—	—	—	—	5.79 (2.36, 17.43)	< **0.01**	4.51 (1.82, 13.74)	< **0.01**
Trauma (16‐53)	—	—	—	—	13.59 (5.65, 40.51)	< **0.01**	8.65 (3.51, 26.18)	< **0.01**
2+ MH/ND conditions^e^	—	—	—	—	—	—	2.80 (1.97, 4.00)	< **0.01**
Daily mental health condition								
Autism diagnosis	5.78 (4.16, 8.10)	< **0.01**	5.94 (4.15, 8.58)	< **0.01**	3.58 (2.42, 5.33)	< **0.01**	—	—
Trauma (6‐10)	—	—	—	—	3.48 (1.65, 7.99)	< **0.01**	—	—
Trauma (11‐15)	—	—	—	—	6.01 (2.84, 13.79)	< **0.01**	—	—
Trauma (16‐53)	—	—	—	—	15.65 (7.42, 35.84)	< **0.01**	—	—
2+ MH/ND conditions^e^	—	—	—	—	—	—	—	—
Substance use to cope								
Autism diagnosis	1.46 (1.08, 1.97)	**0.02**	1.39 (1.01, 1.91)	0.05	0.78 (0.54, 1.12)	0.18	0.71 (0.49, 1.02)	0.08
Trauma (6‐10)	—	—	—	—	4.22 (1.56, 14.82)	**0.01**	3.93 (1.45, 13.84)	**0.02**
Trauma (11‐15)	—	—	—	—	7.08 (2.65, 24.71)	< **0.01**	6.27 (2.33, 21.98)	< **0.01**
Trauma (16‐53)	—	—	—	—	17.31 (6.61, 59.74)	< **0.01**	13.75 (5.16, 47.95)	< **0.01**
2+ MH/ND conditions^e^	—	—	—	—	—	—	1.65 (1.15, 2.37)	**0.01**

*Note:* Values in bold indicate *p* values less than 0.05.

^a^ Model 1 = unadjusted.

^b^ Model 2 = autism and demographics.

^c^ Model 3 = autism, demographics, and traumatic experiences.

^d^ Model 4 = autism, demographics, traumatic experiences, and 2+ co‐occurring mental health and/or neurodevelopmental conditions.

^e^ Mental health and/or neurodevelopmental conditions include: anxiety disorder, language delay, attention deficit hyperactivity disorder (ADHD)/attention deficit disorder (ADD), obsessive compulsive disorder (OCD), oppositional defiant disorder (ODD), bipolar disorder, panic disorder, conduct disorder, personality disorder, depression, post‐traumatic stress disorder, dyslexia, schizophrenia/psychosis, dyspraxia/developmental coordination disorder, sensory processing disorder, eating disorder, social phobia/social anxiety disorder, intellectual disability, specific phobia, generalized anxiety disorder, Tourette syndrome/Tic disorder.

When assessing the association between autistic traits (rather than autism diagnosis) and psychological distress and SRB, the results were highly similar to those reported in Table 2; the only notable difference was that having 6–10 traumatic experiences was not associated with suicide plans in modeling related to autistic traits (Table S2).

### 3.2 Frequency of Co‐Occurrence of Psychological Distress, Suicide‐Related Behaviors, and Trauma

**FIGURE 1:**
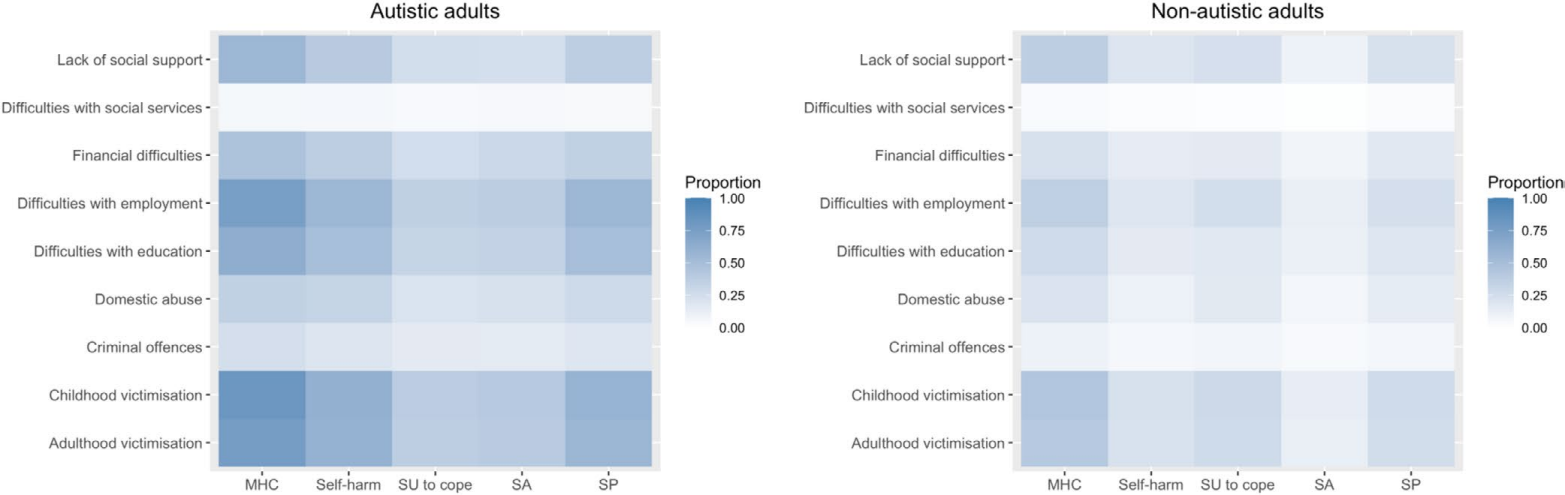
Heatmaps showing the frequency of co‐occurrence of psychological distress, suicide‐related behaviors, and trauma in autistic and non‐autistic adults. MHC = a mental health condition impacting daily life. SU = substance use to cope. SA = suicide attempt. SP = suicide plans.

Figure 1 shows the frequency of co‐occurrence between lifetime traumas and lifetime psychological distress and SRB. Many autistic people who experienced childhood victimization also reported having a mental health condition that impacts daily life. Childhood victimization was also associated with self‐harm and suicide plans in autistic people. Autistic adults who experienced employment‐related traumas also reported a mental health condition that impacts daily life, self‐harm, and suicide plans. Other traumas (including lack of social support, education‐related traumas, and adulthood victimization) were associated with a mental health condition that impacts daily life, self‐harm, and suicide plans in autistic adults. In the non‐autistic group, adulthood and childhood victimization, employment‐related traumas, and lack of social support frequently co‐occurred with having a mental health condition that impacts daily life.

## 4 Discussion

This study aimed, for the first time, to investigate how lifetime trauma and having an autism diagnosis independently relate to lifetime self‐harm, suicide attempts, suicide plans, a mental health condition that impacts daily life, and regularly using substances such as alcohol as a coping mechanism. Irrespective of trauma, autistic adults were still more likely to report lifetime self‐harm, suicide attempts, suicide plans, and a mental health condition that impacts daily life. However, we did not find any significant group differences in the use of substances as coping mechanisms. In both autistic and non‐autistic adults, experiencing high levels of traumatic experiences (16+ traumas) across the lifetime was associated with lifetime psychological distress and SRB. Among autistic adults, childhood victimization and employment‐related traumas were frequently associated with experiencing a mental health condition that impacts daily life, self‐harm, and suicide plans. Moreover, autistic people who experienced a lack of social support, education‐related traumas, and adulthood victimization also reported a mental health condition that impacts daily life, self‐harm, and suicide plans. In non‐autistic people, adulthood and childhood victimization, employment‐related traumas, and lack of social support were frequently associated with a mental health condition that impacts daily life. Taken together, these findings contribute to the limited literature on how trauma is associated with psychological distress and SRB in autistic people (Pelton et al. 2020) and have key clinical, scientific, and policy implications.

Our findings suggest that trauma may have a dose‐dependent effect in our sample. Experiencing the highest levels of lifetime trauma was associated with lifetime psychological distress and SRB in autistic and non‐autistic people. This finding is in line with research in autistic and non‐autistic people reporting suicidality as a result of trauma (Pelton et al. 2020; Ásgeirsdóttir et al. 2018). Although it has been established that autistic people have more traumatic experiences than non‐autistic people (Taylor and Gotham 2016; Griffiths et al. 2019; Pelton et al. 2020), it is not clear whether trauma itself is one of the reasons that autistic people are at an increased risk of suicidality (Brown et al. 2024). This study does not delineate the reasons for lifetime psychological distress and SRB in autistic people. However, the influence of an autism diagnosis on lifetime self‐harm, suicide attempts, suicide plans and a mental health condition that impacts daily life is attenuated by lifetime trauma. This is maintained even after accounting for multiple co‐occurring conditions. These findings provide evidence of complex associations between autism, trauma, neurodevelopmental and mental health conditions, self‐harm, suicide attempts, and suicide plans. It is possible that trauma is partially mediating or moderating the relationship between autism and self‐harm, suicide attempts, suicide plans, and a mental health condition that impacts daily life. However, as this study examined cross‐sectional associations between autism diagnosis, lifetime trauma, psychological distress, and SRB, we could neither identify nor establish the temporal order of the development of either trauma or psychological distress and SRB. Various aspects or traits of autism may contribute to psychological distress and SRB, such as camouflaging, social communication differences, logical approaches, sensory sensitivities, or focused interests and structured routines (Cassidy et al. 2020; Richards et al. 2019; Smith et al. 2023; Stanley et al. 2021; Hedley et al. 2021; Normansell‐Mossa et al. 2021). This aligns with the hypothesized transactional model of trauma in autistic people where autism‐related factors could possibly influence outcomes subsequent to trauma (Kerns et al. 2015). Given that some of these autism‐related factors have been associated with suicidality, for example, camouflaging (Cassidy et al. 2020), further research is needed to better understand the mechanisms that underlie traumatic events, psychological distress, and SRB in autistic people. Clinicians should be aware that higher levels of lifetime trauma are associated with lifetime psychological distress and SRB in autistic and non‐autistic people. However, we do not know how and why autistic people remain at a greater risk of lifetime suicidality compared to non‐autistic people.

Higher levels of trauma (including reporting 6–10, 11–15, or 16+ traumas) were also associated with using substances as a coping mechanism. This is consistent with research suggesting that autistic people may use substances to cope with traumatic experiences (Weir et al. 2021). It has been reported that autistic males are less likely to smoke or use recreational drugs in comparison to non‐autistic males; however, the same study found no differences between autistic and non‐autistic females regarding recreational substance use (Weir et al. 2021). As our sample was predominantly composed of females, this may explain the absence of group differences in substance use. Participants in our community engagement also suggested that some autistic people do not engage in substance use because of their sensory experiences. It is important to acknowledge that research on substance use and abuse in autistic people has produced complex and sometimes conflicting findings (Ressel et al. 2020). More nuanced studies are needed to better understand the relationship between autism and substance use vs. abuse, including potential risk factors and protective mechanisms. Co‐creating and validating new measurement tools may be necessary to accurately capture autistic people's substance‐related experiences.

One of the novel findings of this work is that different types of traumatic experiences are associated with different types of psychological distress and SRB in autistic people. Although existing research in this area is limited for both autistic and general populations, our findings align with one existing paper (Mandell et al. 2005). The previous study found that, compared to children who had not experienced physical or sexual abuse, autistic children that had been physically abused were more likely to attempt suicide, and those that had been sexually abused were more likely to attempt suicide, engage in self‐injurious behavior, and experience suicide‐related problems (Mandell et al. 2005). While this study considers a more limited range of trauma with SRB, it also illustrates that different types of trauma may be associated with different types of SRB among autistic people.

Our data demonstrates that there is a frequent co‐occurrence between childhood victimization and a mental health condition that impacts daily life, self‐harm, and suicide plans in both the autistic and non‐autistic groups, with approximately 60%–80% of autistic people reporting both compared to approximately 20%–40% of non‐autistic people. Childhood traumas, such as poverty, sexual abuse, physical abuse, school exclusion, bullying, insults, and humiliation, are particularly common among autistic people (Griffiths et al. 2019; Hoover and Kaufman 2018). This may be partly attributed to stigma and lack of acceptance (Botha et al. 2022). Autistic adults in our community engagement focus group expressed feeling a *backlog* of childhood traumas that significantly impact their adult lives. Future research should explore whether similar traumas across different life stages (e.g., peer victimization at work versus in school) amplify the harmful effects of trauma in autistic adults. In addition, it should address how childhood traumas may influence the progression from suicidal ideation to plans and attempts in autistic adults. We also identified that adulthood victimization frequently co‐occurs with a mental health condition that affects day‐to‐day life, self‐harm, and suicide plans in autistic people. It has been established that autistic adults are at higher risk of bullying, physical abuse, sexual abuse than non‐autistic adults (Griffiths et al. 2019; Weiss and Fardella 2018; Douglas and Sedgewick 2024). However, we need further research to understand how these individual traumas in adulthood relate to different types of psychological distress and SRB.

Autistic people who reported not having support (during trouble or illness) or not knowing someone loves them, also frequently reported a mental health condition that impacts daily life, self‐harm, and suicide plans. Previous research has shown that unmet support needs significantly predict suicidality in autistic people (Cassidy et al. 2018). Therefore, there is an urgent need to identify barriers in support provision in both community and clinical settings. Our findings also suggest a complex association between autism, employment, and education. While this study does not provide statistical validation for this association, autistic people have high rates of job loss and protracted periods of unemployment (Griffiths et al. 2019); they are also more likely to drop out of school, miss school because of depression or anxiety, experience school exclusion, lack additional school support, and avoid school lessons because of stress compared to non‐autistic people (Griffiths et al. 2019). Despite employment schemes and special educational needs‐based support to help autistic people, current efforts appear insufficient. Promotion of good mental health within educational settings and workplaces could be improved through training on neurodiversity and autism, as well as autism‐friendly working conditions, such as accommodating office spaces to sensory sensitivities (de Vries 2021).

Given that we see different types of traumas relating to different types of psychological distress and SRB, future research should investigate whether certain traumas are differentially associated with suicidal ideation, plans, and attempts. On a theoretical level, further studies should also explore whether trauma is conceptualized and experienced the same way between autistic and non‐autistic people, as there are several barriers to understanding trauma in autistic people and offering appropriate support. First, there is a potential to misattribute trauma‐related symptoms to core features of autism (Brenner et al. 2018). For example, autistic traits, such as strong interests and a preference for routine, may overlap with trauma‐related behaviors such as repetitive play used to express traumatic events (DSM‐5 2013). Second, trauma is related to anxiety and depression in autistic people (Taylor and Gotham 2016; Griffiths et al. 2019) which could complicate the diagnostic process and provision of appropriate support based on their needs. Thus, the overlap of trauma symptoms with mental health conditions may complicate assessments and interventions. Third, autistic people have expressed that they experience trauma throughout their lifetime but learn various coping mechanisms to promote resilience (Ghanouni and Quirke 2023). This makes it particularly difficult to identify which specific traumas may underlie psychological distress and SRB. Understanding how different traumas are associated with different types of psychological distress and SRB can be critical in designing early interventions to address these outcomes.

It is crucial to understand suicide progression in autistic people (e.g., from making a plan to making an attempt at ending their life), so that we can identify how to prevent suicide risk from progressing. Although mapping different lifetime traumas to lifetime psychological distress and SRB does not give a clear pathway of how and why suicidality occurs in autistic people, it highlights the areas that require urgent intervention, such as childhood victimization. For example, our finding that childhood victimization is frequently associated with a mental health condition that impacts daily life, self‐harm, and suicide plans highlights the need to raise awareness about autism and child abuse, train relevant staff to identify child maltreatment, establish clear reporting pathways for concerns, and provide equal access to services. Additionally, co‐creation of safeguarding strategies with clinicians, autistic people, and policymakers for addressing traumatic events affecting autistic children may be important suicide prevention tools for autistic people.

Our findings suggest that suicidality should not only be studied as a single construct, as we need to understand how one transitions from suicidal plans to thoughts to behaviors. Similarly, trauma should not be viewed or treated in a monolithic way. In the general population, it has been established that not everyone who experiences suicidal ideation will plan or attempt suicide (Klonsky and May 2014). Contrary to existing literature, we do not know whether these patterns also apply to the autistic population. Pelton and colleagues suggest that sometimes trauma can happen in the absence of lifetime suicidality in autistic people (Pelton et al. 2020). It may be possible that the impact of trauma on suicidality may vary from person to person in the autism community. Our understanding of trauma and suicide in autistic people, although important, remains limited. The Interpersonal Psychological Theory of Suicide (IPTS) has been studied in autistic and non‐autistic people; the theory hypothesizes that repeated lifetime exposure to negative experiences lowers the fear of death, making suicidal behavior more likely; but this was not supported by the findings in the Pelton et al. study (Pelton et al. 2020). While the IPTS has been explored in autistic people (Pelton et al. 2020), future research should consider the application of other existing suicide theories such as the three‐step theory (Klonsky et al. 2021). Coupled with the emerging knowledge about how trauma relates to suicide and existing suicide risk factors (Pelton et al. 2020; Brown et al. 2024; Cassidy et al. 2014), further studies should investigate how dispositional and/or practical factors (such as sensory experiences) (MacLennan et al. 2022), emotion dysregulation (Conner et al. 2021), or logical thinking (Brosnan et al. 2016) contribute to psychological distress and SRB in autistic people.

This study has vital clinical and scientific implications. Autistic people report feeling misunderstood by mental health professionals and communicate a desire for autism‐specific support (Camm‐Crosbie et al. 2019). Given the vulnerability of autistic people to a range of traumatic experiences (Taylor and Gotham 2016; Griffiths et al. 2019; Moseley et al. 2021), mental health specialists should be aware of how to differentiate between trauma‐related symptoms and autistic traits. Training programmes for mental health professionals on autism and trauma may also be valuable, as taking a trauma‐informed approach for mental health care of autistic people may often be appropriate. Second, autistic people, researchers, clinicians, educators, employers, and social care professionals should work together to develop safeguarding strategies that appropriately address childhood victimization, as well as tailored supports for financial or employment difficulties for autistic people. Third, given that childhood victimization frequently co‐occurs with lifetime psychological distress and SRB in autistic people, further research is needed to identify which specific childhood traumas may lead to psychological distress and SRB later in life. In addition, future research must investigate other autism‐specific risk factors for psychological distress and SRB.

This study has several strengths and limitations. Notably, it is the first large‐scale study to investigate multiple lifetime traumas in relation to lifetime self‐harm, suicide attempts, suicide plans, mental health conditions impacting daily life, and substance use as a coping mechanism in autistic people. Our study is also the first to identify different types of lifetime traumas that frequently co‐occur with different types of psychological distress and SRB in autistic adults. Additionally, the study employed a participatory approach (Nicolaidis et al. 2011), ensuring that measures were appropriate for autistic people and prioritized autistic people's research interests. There are also some limitations that should be noted. First, our sample size is relatively small and is not representative of autistic or non‐autistic people generally. Our sample is mostly female, highly educated, and only included people able to fill in a lengthy, online survey; as such, it is unlikely to represent the experiences of all autistic people. These findings may underestimate the challenges faced by some autistic people, such as those with relatively lower socioeconomic status and/or co‐occurring intellectual disability. In addition, the sample is likely subject to sampling biases due to the methods of collection, as autistic people or those with an interest in autism, as well as individuals with relatively high levels of trauma may be more likely to have participated in the study. Limitations of sampling should be addressed in future studies. Second, despite including different types of lifetime psychological distress and SRB, our study has not explored suicidal ideation as an outcome variable which is an important aspect in the suicide ideation‐to‐action frameworks. Additionally, it is possible that the measure for lifetime trauma employed in this study under‐reported the negative experiences autistic people have. We used the VEQ which was co‐created several years ago with autistic adults; however, this produces a binary outcome which does not consider temporality, length of exposure, repeatability, or severity of the traumatic experiences. These aspects are what make trauma complex and subjective; these complexities often shape people's experiences and, ultimately, their mental health outcomes.

More work is needed to understand how different traumas influence suicidal ideation and attempts considering the mechanisms that lead to the development and maintenance of trauma. Future work should also assess the relationships between age of autism diagnosis, misdiagnosis, trauma, psychological distress, and SRB. Fourth, our analyses are based on cross‐sectional data; however, future studies could adopt a longitudinal approach to track the impact of different types of trauma on psychological distress and SRB while considering severity. Finally, the study has not explored lifetime depression and anxiety. Previous studies have shown that autistic people are more likely to report higher levels of anxiety and depression symptoms (Griffiths et al. 2019; Lever and Geurts 2016; Pelton et al. 2023). There is also evidence of trauma partially influencing anxiety and depression symptoms in autistic people (Griffiths et al. 2019). Future studies should look at the associations between lifetime depression and anxiety, trauma, neurodevelopmental conditions, psychological distress, and SRB in autistic people.

In summary, this study is the first to show that specific types of lifetime traumas are associated with different forms of lifetime psychological distress and SRB. This study shows that autistic people are more likely to report self‐harm, suicide attempts, suicide plans, and a mental health condition that impacts daily life, even after accounting for several traumatic experiences and multiple co‐occurring conditions. Clinicians and policymakers should be aware of the association between lifetime trauma and lifetime psychological distress and SRB in autistic people. It is important for clinicians to record detailed histories of autistic people's experiences and to provide needs‐based support. Our findings emphasize the need for autistic people, researchers, clinicians, educators, employers, and social care professionals to ensure that appropriate safeguarding measures are put in place in educational settings, workplaces, and communities. Overall, there is an urgent need to develop and implement tailored programmes that prevent psychological distress and SRB in autistic people.

## Author Contributions

**T.A.C.:** writing – review and editing, writing – original draft, formal analysis, methodology, data curation, visualization. **E.W.:** writing – review and editing, conceptualization, methodology, supervision. **S.G.:** data collection, methodology, funding acquisition. **T.P.:** writing – review and editing. **M.P.:** writing – review and editing. **C.A.:** writing – review and editing, funding acquisition, methodology. **H.H.:** writing – review and editing. **S.R.W.:** writing – review and editing, methodology. **T.F.:** writing – review and editing, supervision. **S.B.‐C.:** writing – review and editing, funding acquisition, methodology.

## Conflicts of Interest

The authors declare no conflicts of interest.

## Supporting information


**Data S1:** aur70137‐sup‐0001‐Supinfo.docx.

## Data Availability

As participants did not consent for their data to be publicly shared, even anonymized, data will be made available to only potential collaborators with ethical approval after they submit a research proposal to the Autism Research Centre, University of Cambridge, UK.
