# Supplementary material for: Traumatic Experiences, Psychological Distress and Suicide‐Related Behaviors in Autistic Adults
Source: Autism Res. 2025 Nov 25;19(1):e70137. doi: 10.1002/aur.70137 (PMC12853244; doi:10.1002/aur.70137)
Supplement: Supplementary file 1 — Data S1: aur70137‐sup‐0001‐Supinfo.docx. [file AUR-19-0-s001.docx]

**Supplementary Materials**

1. Supplementary item 1: Vulnerability Experiences Quotient (VEQ)
2. Supplementary item 2 VEQ mental health difficulties subdomain statements
3. Supplementary item 3 Autism Spectrum Quotient (AQ-10)
4. Supplementary item 4: Logistic regression exploring the association between traumatic experiences and self-harm, suicide attempts, suicide plans, a daily mental health condition, and substance use to cope in autistic and non-autistic adults
5. Supplementary item 5: Logistic regression exploring the association between autistic traits, traumatic experiences, self-harm, suicide attempts, suicide plans, a daily mental health condition, and substance use to cope

**Supplementary item 1** Vulnerability Experiences Quotient (VEQ)

The VEQ is a 60-item scale that presents negative life events across 10 thematic areas which include difficulties with education, difficulties with employment, financial difficulties, difficulties with social services, criminal offence, childhood and adulthood victimisation, domestic abuse, lack of social support, and mental health. Each area has between three and nine statements, and they are presented in a random order. Most of the statements in the VEQ are negatively worded, except for three social support questions that were included to avoid a complete focus on negative experiences. These social support questions include statements such as “*There has always been someone in my life who would care for me if I was ill*.” Participants were asked to respond to each item by selecting “yes”, “no”, or “no opportunity.” Participants’ responses were coded as 1 for “yes” and 0 for “no” or “no opportunity” on all items except for the three social support that were reverse scored indicating 0 for “yes” and 1 for “no” or “no opportunity” (1).

**Supplementary item 2** VEQ mental health difficulties subdomain statements

*Self-harm, suicide attempt, suicide plans, a daily mental health condition, and substance use to cope*] were assessed with the following statements:

1. *I have deliberately harmed myself*
2. *I have attempted suicide*
3. *I have made suicide plans*
4. *I have had a mental health condition that affected my daily life*
5. *There was a period of my life where I was regularly using alcohol or another (non-prescribed) drug in order to cope*.

**Supplementary item 3** Autism Spectrum Quotient (AQ-10)

The AQ-10 is inclusive of 10-items from the original Autism-Spectrum Quotient (AQ) (2) which was quantitatively developed to measure autistic traits in the general public. Participants are asked how much they agree/disagree with 10 statements about themselves (e.g. I often notice small sounds when others do not). Participants can either choose: “definitely agree”, “slightly agree”, “slightly disagree” or “definitely disagree”. “Definitely agree” and “slightly agree” receive a score of 0 while “slightly disagree” and “definitely disagree” receive a score of 1. There are four items that are inversely scored. A score on the AQ-10 of six or above indicates elevated autistic traits that may warrant a clinical diagnostic assessment.

**Supplementary item 4**

***Table 1*** *Logistic regression exploring the association between traumatic experiences and self-harm, suicide attempts, suicide plans, a daily mental health condition, and substance use to cope in autistic and non-autistic adults*

|  | **Model 1^a^** | | **Model 2^b^** | | **Model 3^c^** | | **Model 4^d^** | |
| --- | --- | --- | --- | --- | --- | --- | --- | --- |
|  | Odds Ratio (95% CI) | p-value | Odds Ratio (95% CI) | p-value | Odds Ratio (95% CI) | p-value | Odds Ratio (95% CI) | p-value |
|  | **Self-harm** | | | | | | | |
| Autism Diagnosis | 4.90  (3.59, 6.73) | **<0.01** | 4.83  (3.43, 6.87) | **<0.01** | 2.71  (1.85, 4.00) | **<0.01** | 2.36  (1.59, 3.52) | **<0.01** |
| Age | - | **-** | 0.96  (0.94, 0.97) | **<0.01** | 0.95  (0.94, 0.96) | **<0.01** | 0.95  (0.94, 0.97) | **<0.01** |
| Male sex | - | **-** | 0.52  (0.36, 0.74) | **<0.01** | 0.59  (0.40, 0.86) | **0.01** | 0.65  (0.43, 0.96) | **0.03** |
| Other country | - | **-** | 0.85  (0.54, 1.34) | 0.49 | 0.90  (0.55, 1.47) | 0.67 | 0.98  (0.60, 1.62) | 0.95 |
| United States of America | - | **-** | 0.77  (0.48, 1.22) | 0.26 | 0.68  (0.41, 1.11) | 0.13 | 0.61  (0.37, 1.01) | 0.06 |
| Further vocational qualifications | - | **-** | 0.72  (0.41, 1.27) | 0.26 | 0.48  (0.26, 0.89) | **0.02** | 0.48  (0.25, 0.89) | **0.02** |
| University undergraduate level qualification | - | **-** | 0.51  (0.30, 0.86) | **0.01** | 0.50  (0.28, 0.88) | **0.02** | 0.59  (0.34, 1.02) | **0.01** |
| University post-graduate level qualification | - | **-** | 0.58  (0.35, 0.94) | **0.03** | 0.58  (0.34, 0.99) | 0.05 | 0.49  (0.27, 0.86) | 0.06 |
| Trauma (6-10) | - | - | - | - | 1.87  (0.64, 6.83) | 0.29 | 1.65  (0.57, 6.06) | 0.40 |
| Trauma (11-15) | - | - | - | - | 4.56  (1.66, 16.14) | **0.01** | 3.56  (1.28, 12.70) | **0.03** |
| Trauma (16-53) | - | - | - | - | 16.80  (6.28, 58.75) | **<0.01** | 11.06  (4.04, 39.15) | **<0.01** |
| 2+ MH/ND conditions^e^ | - | - | - | - | - | - | 2.40  (1.63, 3.53) | **<0.01** |
|  | **Suicide attempts** | | | | | | | |
| Autism Diagnosis | 4.29  (3.02, 6.19) | **<0.01** | 4.23  (2.93, 6.21) | **<0.01** | 2.45  (1.65, 3.68) | **<0.01** | 2.08  (1.38, 3.16) | **<0.01** |
| Age | - | **-** | 1.00  (0.98, 1.01) | 0.40 | 1.00  (0.98, 1.01) | 0.58 | 1.00  (0.99, 1.02) | 0.82 |
| Male sex | - | **-** | 0.73  (0.51, 1.04) | 0.08 | 0.84  (0.57, 1.22) | 0.36 | 0.94  (0.64, 1.39) | 0.77 |
| Other country | - | **-** | 0.76  (0.47, 1.19) | 0.24 | 0.77  (0.47, 1.25) | 0.30 | 0.85  (0.51, 1.39) | 0.51 |
| United States of America | - | **-** | 0.80  (0.49, 1.28) | 0.37 | 0.71  (0.42, 1.16) | 0.18 | 0.61  (0.36, 1.01) | 0.06 |
| Further vocational qualifications | - | **-** | 0.81  (0.47, 1.38) | 0.43 | 0.63  (0.36, 1.11) | 0.11 | 0.63  (0.35, 1.12) | 0.11 |
| University undergraduate level qualification | - | **-** | 0.61  (0.37, 1.00) | 0.05 | 0.64  (0.38, 1.08) | 0.10 | 0.62  (0.37, 1.05) | 0.08 |
| University post-graduate level qualification | - | **-** | 0.61  (0.38, 0.99) | **0.04** | 0.61  (0.37, 1.02) | 0.06 | 0.61  (0.36, 1.05) | 0.08 |
| Trauma (6-10) | - | - | - | - | 5.80  (1.13, 106.24) | 0.10 | 4.82  (0.93, 88.70) | 0.14 |
| Trauma (11-15) | - | - | - | - | 8.53  (1.73, 154.65) | 0.05 | 6.13  (1.22, 111.67) | 0.09 |
| Trauma (16-53) | - | - | - | - | 31.99  (6.74, 573.18) | **<0.01** | 18.93  (3.89, 341.51) | **0.01** |
| 2+ MH/ND conditions^e^ | - | - | - | - | - | - | 3.01  (2.01, 4.56) | **<0.01** |
|  | **Suicide plans** | | | | | | | |
| Autism Diagnosis | 3.52  (2.61, 4.78) | **<0.01** | 3.34  (2.44, 4.59) | **<0.01** | 2.00  (1.41, 2.83) | **<0.01** | 1.68  (1.17, 2.40) | **0.01** |
| Age | - | **-** | 0.99  (0.98, 1.00) | **0.02** | 0.99  (0.98, 1.00) | 0.11 | 0.99  (0.98, 1.01) | 0.34 |
| Male sex | - | **-** | 0.77  (0.55, 1.06) | 0.11 | 0.87  (0.62, 1.22) | 0.42 | 0.99  (0.69, 1.41) | 0.95 |
| Other country | - | **-** | 0.94  (0.62, 1.42) | 0.75 | 0.95  (0.62, 1.47) | 0.82 | 1.03  (0.66, 1.60) | 0.90 |
| United States of America | - | **-** | 0.70  (0.46, 1.07) | 0.10 | 0.60  (0.38, 0.93) | **0.02** | 0.52  (0.32, 0.81) | **<0.01** |
| Further vocational qualifications | - | **-** | 1.02  (0.61, 1.71) | 0.93 | 0.83  (0.49, 1.42) | 0.50 | 0.81  (0.47, 1.41) | 0.46 |
| University undergraduate level qualification | - | **-** | 0.88  (0.55, 1.40) | 0.58 | 0.93  (0.57, 1.52) | 0.77 | 0.90  (0.54, 1.49) | 0.68 |
| University post-graduate level qualification | - | **-** | 0.88  (0.57, 1.38) | 0.58 | 0.91  (0.57, 1.46) | 0.71 | 0.94  (0.58, 1.53) | 0.81 |
| Trauma (6-10) | - | - | - | - | 2.92  (1.16, 8.93) | **0.04** | 2.54  (1.00, 7.81) | 0.08 |
| Trauma (11-15) | - | - | - | - | 5.79  (2.36, 17.43) | **<0.01** | 4.51  (1.82, 13.74) | **<0.01** |
| Trauma (16-53) | - | - | - | - | 13.59  (5.65, 40.51) | **<0.01** | 8.65  (3.51, 26.18) | **<0.01** |
| 2+ MH/ND conditions^e^ | - | - | - | - | - | - | 2.80  (1.97, 4.00) | **<0.01** |
|  | **Daily mental health condition** | | | | | | | |
| Autism Diagnosis | 5.78  (4.16, 8.10) | **<0.01** | 5.94  (4.15, 8.58) | **<0.01** | 3.58  (2.42, 5.33) | **<0.01** | - | **-** |
| Age | - | **-** | 0.97  (0.96, 0.98) | **<0.01** | 0.98  (0.96, 0.99) | **<0.01** | - | **-** |
| Male sex | - | **-** | 0.53  (0.36, 0.76) | **<0.01** | 0.54  (0.37, 0.81) | **<0.01** | - | **-** |
| Other country | - | **-** | 0.58  (0.37, 0.93) | **0.02** | 0.55  (0.34, 0.90) | **0.02** | - | **-** |
| United States of America | - | **-** | 0.92  (0.59, 1.45) | 0.72 | 0.76  (0.48, 1.23) | 0.27 | - | **-** |
| Further vocational qualifications | - | **-** | 1.95  (1.06, 3.62) | **0.03** | 1.67  (0.87, 3.24) | 0.13 | - | **-** |
| University undergraduate level qualification | - | **-** | 1.03  (0.60, 1.75) | 0.91 | 1.10  (0.62, 1.92) | 0.75 | - | **-** |
| University post-graduate level qualification | - | **-** | 1.05  (0.64, 1.74) | 0.84 | 1.12  (0.66, 1.91) | 0.67 | - | **-** |
| Trauma (6-10) | - | - | - | - | 3.48  (1.65, 7.99) | **<0.01** | - | **-** |
| Trauma (11-15) | - | - | - | - | 6.01  (2.84, 13.79) | **<0.01** | - | **-** |
| Trauma (16-53) | - | - | - | - | 15.65  (7.42, 35.84) | **<0.01** | - | **-** |
| 2+ MH/ND conditions^e^ | - | - | - | - | - | - | - | **-** |
|  | **Substance use to cope** | | | | | | | |
| Autism Diagnosis | 1.46  (1.08, 1.97) | 0.02 | 1.39  (1.01, 1.91) | 0.05 | 0.78  (0.54, 1.12) | 0.18 | 0.71  (0.49, 1.02) | 0.08 |
| Age | - | - | 1.00  (0.99, 1.01) | 0.85 | 1.00  (0.99, 1.02) | 0.50 | 1.01  (0.99, 1.02) | 0.31 |
| Male sex | - | - | 0.89  (0.64, 1.23) | 0.47 | 0.98  (0.70, 1.38) | 0.91 | 1.04  (0.74, 1.47) | 0.82 |
| Other country | - | - | 0.70  (0.45, 1.07) | 0.10 | 0.68  (0.43, 1.05) | 0.09 | 0.70  (0.44, 1.10) | 0.13 |
| United States of America | - | - | 0.82  (0.53, 1.24) | 0.35 | 0.69  (0.44,1.07) | 0.11 | 0.65  (0.41, 1.02) | 0.06 |
| Further vocational qualifications | - | - | 0.86  (0.53, 1.41) | 0.56 | 0.69  (0.41, 1.16) | 0.16 | 0.69  (0.41, 1.15) | 0.15 |
| University undergraduate level qualification | - | - | 0.65  (0.41, 1.02) | 0.06 | 0.65  (0.41, 1.05) | 0.08 | 0.64  (0.40, 1.04) | 0.07 |
| University post-graduate level qualification | - | - | 0.46  (0.30, 0.72) | **<0.01** | 0.44  (0.28, 0.69) | **<0.01** | 0.44  (0.28, 0.70) | **<0.01** |
| Trauma (6-10) | - | - | - | - | 4.22  (1.56, 14.82) | **0.01** | 3.93  (1.45, 13.84) | **0.02** |
| Trauma (11-15) | - | - | - | - | 7.08  (2.65, 24.71) | **<0.01** | 6.27  (2.33, 21.98) | **<0.01** |
| Trauma (16-53) | - | - | - | - | 17.31  (6.61, 59.74) | **<0.01** | 13.75 (5.16, 47.95) | **<0.01** |
| 2+ MH/ND conditions^e^ | - | - | - | - | - | - | 1.65  (1.15, 2.37) | **0.01** |

Note. p-values for the following variables were adjusted for multiple testing correction: autism, trauma, 2+MH/ND conditions. Values in bold indicate p-values less than 0.05.

^a^Model 1 = unadjusted. ^b^Model 2 = autism and demographics. ^c^Model 3 = autism, demographics, and traumatic experiences. ^d^Model 4 = autism, demographics, traumatic experiences, and two or more co-occurring mental health and/or neurodevelopmental conditions.^e^Mental health and/or neurodevelopmental conditions include: Anxiety disorder, Language delay, Attentional deficit hyperactivity disorder (ADHD)/Attentional deficit disorder (ADD), Obsessive compulsive disorder (OCD), Oppositional defiant disorder (ODD), Bipolar disorder, Panic disorder, Conduct disorder, Personality disorder, Depression, Post­traumatic stress disorder, Dyslexia, Schizophrenia/Psychosis, Dyspraxia/Developmental coordination disorder, Sensory processing disorder, Eating disorder, Social phobia/Social anxiety disorder, Intellectual disability, Specific phobia, Generalised anxiety disorder, Tourette syndrome/Tic disorder.

**Supplementary item 5**

***Table 2*** *Logistic regression exploring the association between autistic traits, traumatic experiences, self-harm, suicide attempts, suicide plans, a daily mental health condition, and substance use to cope*

After accounting for trauma and demographic differences, high autistic traits were associated with self-harm (OR: 2.09, 95% CI: 1.43, 3.07, p-value: <0.01), suicide attempts (OR: 1.88, 95% CI: 1.27, 2.80, p-value: <0.01), suicide plans (OR: 2.16, 95% CI: 1.53, 3.05, p-value: <0.01), and a mental health condition that affects day-to-day lives (OR: 3.02, 95% CI: 2.06, 4.44, p-value: <0.01). High autistic traits were also associated with self-harm (OR: 1.87, 95% CI: 1.26, 2.77, p-value: <0.01), suicide attempts (OR: 1.69, 95% CI: 1.13, 2.53, p-value: 0.02), and suicide plans (OR: 1.93, 95% CI: 1.36, 2.76, p-value: <0.01) even after accounting for trauma and multiple co-occurring mental health and/or neurodevelopmental conditions. Irrespective of trauma and multiple co-occurring conditions, autistic traits were not associated with the use of substances such as alcohol to cope.

|  | **Model 1^a^** | | **Model 2^b^** | | **Model 3^c^** | | **Model 4^d^** | |
| --- | --- | --- | --- | --- | --- | --- | --- | --- |
|  | Odds Ratio (95% CI) | p-value | Odds Ratio (95% CI) | p-value | Odds Ratio (95% CI) | p-value | Odds Ratio (95% CI) | p-value |
|  | **Self-harm** | | | | | | | |
| High AQ Score | 3.87  (2.85, 5.29) | **<0.01** | 3.90  (2.78, 5.50) | **<0.01** | 2.09  (1.43, 3.07) | **<0.01** | 1.87  (1.26, 2.77) | **<0.01** |
| Age | - | - | 0.95  (0.94, 0.96) | **<0.01** | 0.95  (0.94, 0.96) | **<0.01** | 0.95  (0.94, 0.96) | **<0.01** |
| Male sex | - | - | 0.58  (0.41, 0.82) | **<0.01** | 0.65  (0.45, 0.95) | **0.02** | 0.72  (0.49, 1.05) | 0.09 |
| Other country | - | - | 0.85  (0.55, 1.33) | 0.48 | 0.89  (0.55, 1.45) | 0.64 | 0.99  (0.60, 1.61) | 0.96 |
| United States of America | - | - | 0.79  (0.50, 1.24) | 0.31 | 0.66  (0.40, 1.08) | 0.10 | 0.60  (0.36, 0.98) | **0.04** |
| Further vocational qualifications | - | - | 0.73  (0.42, 1.27) | 0.27 | 0.47  (0.25, 0.87) | **0.02** | 0.46  (0.25, 0.86) | **0.02** |
| University undergraduate level qualification | - | - | 0.52  (0.31, 0.87) | **0.01** | 0.50  (0.28, 0.87) | **0.01** | 0.48  (0.27, 0.85) | **0.01** |
| University post-graduate level qualification | - | - | 0.54  (0.33, 0.87) | **0.01** | 0.54  (0.32, 0.92) | **0.02** | 0.56  (0.32, 0.96) | **0.04** |
| Trauma (6-10) | - | - | - | - | 1.85  (0.64, 6.74) | 0.31 | 1.61  (0.56, 5.90) | 0.43 |
| Trauma (11-15) | - | - | - | - | 4.98  (1.83, 17.60) | **0.01** | 3.80  (1.38, 13.52) | **0.02** |
| Trauma (16-53) | - | - | - | - | 18.33  (6.84, 64.12) | **<0.01** | 11.58  (4.23, 41.01) | **<0.01** |
| 2+ MH/ND conditions^e^ | - | - | - | - | - | - | 2.55  (1.74, 3.74) | **<0.01** |
|  | **Suicide attempts** | | | | | | | |
| High AQ Score | 3.49  (2.46, 5.01) | **<0.01** | 3.36  (2.35, 4.88) | **<0.01** | 1.88  (1.27, 2.80) | **<0.01** | 1.69  (1.13, 2.53) | **0.02** |
| Age | - | - | 0.99  (0.98, 1.00) | 0.11 | 0.99  (0.98, 1.01) | 0.33 | 1.00  (0.99, 1.01) | 0.99 |
| Male sex | - | - | 0.81  (0.57, 1.14) | 0.23 | 0.92  (0.63, 1.33) | 0.65 | 1.03  (0.70, 1.50_ | 0.89 |
| Other country | - | - | 0.77  (0.48, 1.20) | 0.26 | 0.77  (0.47, 1.25) | 0.30 | 0.86  (0.52, 1.41) | 0.56 |
| United States of America | - | - | 0.81  (0.50, 1.29) | 0.39 | 0.69  (0.41, 1.13) | 0.15 | 0.59  (0.35, 0.98) | 0.05 |
| Further vocational qualifications | - | - | 0.82  (0.48, 1.39) | 0.46 | 0.62  (0.35, 1.08) | 0.09 | 0.62  (0.35, 1.09) | 0.10 |
| University undergraduate level qualification | - | - | 0.62  (0.38, 1.02) | 0.06 | 0.64  (0.38, 1.08) | 0.09 | 0.62  (0.36, 1.05) | 0.08 |
| University post-graduate level qualification | - | - | 0.58  (0.36, 0.93) | **0.02** | 0.59  (0.35, 0.97) | **0.04** | 0.60  (0.36, 1.01) | 0.06 |
| Trauma (6-10) | - | - | - | - | 5.87  (1.14, 107.53) | 0.10 | 4.76  (0.91, 87.51) | 0.15 |
| Trauma (11-15) | - | - | - | - | 9.57  (1.94, 173.39) | **0.04** | 6.66  (1.33, 121.25) | 0.08 |
| Trauma (16-53) | - | - | - | - | 36.12  (7.60, 647.50) | **<0.01** | 20.31  (4.17, 366.61) | **0.01** |
| 2+ MH/ND conditions^e^ | - | - | - | - | - | - | 3.20  (2.14, 4.83) | **<0.01** |
|  | **Suicide plans** | | | | | | | |
| High AQ Score | 3.72  (2.74, 5.07) | **<0.01** | 3.54  (2.59, 4.87) | **<0.01** | 2.16  (1.53, 3.05) | **<0.01** | 1.93  (1.36, 2.76) | **<0.01** |
| Age | - | - | 0.99  (0.97, 1.00) | **0.01** | 0.99  (0.98, 1.00) | 0.07 | 0.99  (0.98, 1.01) | 0.29 |
| Male sex | - | - | 0.80  (0.58, 1.10) | 0.17 | 0.89  (0.63, 1.25) | 0.50 | 1.00  (0.70, 1.42) | 0.99 |
| Other country | - | - | 0.94  (0.62, 1.43) | 0.77 | 0.95  (0.62, 1.47) | 0.83 | 1.04  (0.67, 1.61) | 0.88 |
| United States of America | - | - | 0.73  (0.47, 1.11) | 0.14 | 0.61  (0.39, 0.96) | **0.03** | 0.53  (0.33, 0.84) | **0.01** |
| Further vocational qualifications | - | - | 1.03  (0.62, 1.72) | 0.91 | 0.83  (0.48, 1.42) | 0.49 | 0.81  (0.47, 1.41) | 0.47 |
| University undergraduate level qualification | - | - | 0.88  (0.55, 1.30) | 0.60 | 0.93  (0.57, 1.52) | 0.76 | 0.90  (0.54, 1.49) | 0.67 |
| University post-graduate level qualification | - | - | 0.83  (0.53, 1.30) | 0.42 | 0.88  (0.55, 1.40) | 0.58 | 0.91  (0.56, 1.48) | 0.71 |
| Trauma (6-10^)^ | - | - | - | - | 2.73  (1.08, 8.38) | 0.06 | 2.34  (0.92, 7.24) | 0.11 |
| Trauma (11-15) | - | - | - | - | 5.65  (2.30, 17.03) | **<0.01** | 4.28  (1.72, 13.05) | **0.01** |
| Trauma (16-53) | - | - | - | - | 12.74  (5.28, 38.03) | **<0.01** | 7.78  (3.14, 23.63) | **<0.01** |
| 2+ MH/ND conditions^e^ | - | - | - | - | - | - | 2.85  (2.01, 4.07) | **<0.01** |
|  | **Daily mental health condition** | | | | | | | |
| High AQ Score | 4.92  (3.57, 6.82) | **<0.01** | 5.10  (3.61, 7.28) | **<0.01** | 3.02  (2.06, 4.44) | **<0.01** | - | - |
| Age | - | - | 0.97  (0.96, 0.98) | **<0.01** | 0.97  (0.96, 0.99) | **<0.01** | - | **-** |
| Male sex | - | - | 0.58  (0.40, 0.83) | **<0.01** | 0.59  (0.40, 0.86) | **0.01** | - | **-** |
| Other country | - | - | 0.57  (0.36, 0.91) | **0.02** | 0.53  (0.33, 0.87) | **0.01** | - | **-** |
| United States of America | - | - | 0.96  (0.62, 1.51) | 0.86 | 0.77  (0.48, 1.25) | 0.29 | - | **-** |
| Further vocational qualifications | - | - | 1.94  (1.06, 3.58) | 0.03 | 1.67  (0.88, 3.22) | 0.12 | - | **-** |
| University undergraduate level qualification | - | - | 1.02  (0.60, 1.72) | 0.95 | 1.07  (0.61, 1.87) | 0.80 | - | **-** |
| University post-graduate level qualification | - | - | 0.94  (0.57, 1.55) | 0.82 | 1.04  (0.61, 1.76) | 0.88 | - | **-** |
| Trauma (6-10) | - | - | - | - | 3.27  (1.55, 7.47) | **<0.01** | - | **-** |
| Trauma (11-15) | - | - | - | - | 6.29  (2.99, 14.37) | **<0.01** | - | **-** |
| Trauma (16-53) | - | - | - | - | 15.83  (7.50, 36.27) | **<0.01** | - | **-** |
| 2+ MH/ND conditions^e^ | - | - | - | - | - | **-** | - | **-** |
|  | **Substance use to cope** | | | | | | | |
| High AQ Score | 1.79  (1.32, 2.45) | <0.01 | 1.78  (1.29, 2.45) | **<0.01** | 1.06  (0.74, 1.51) | 0.75 | 1.00  (0.70, 1.43) | 0.99 |
| Age | - | - | 1.00  (0.99, 1.01) | 0.95 | 1.01  (0.99, 1.02) | 0.35 | 1.01  (1.00, 1.02) | 0.20 |
| Male sex | - | - | 0.87  (0.63, 1.20) | 0.40 | 0.93  (0.66, 1.31) | 0.69 | 0.97  (0.69, 1.37) | 0.88 |
| Other country | - | - | 0.70  (0.45, 1.08) | 0.11 | 0.69  (0.43, 1.07) | 0.10 | 0.71  (0.45, 1.10) | 0.13 |
| United States of America | - | - | 0.85  (0.55, 1.29) | 0.45 | 0.72  (0.46, 1.12) | 0.15 | 0.69  (0.43, 1.07) | 0.10 |
| Further vocational qualifications | - | - | 0.86  (0.52, 1.40) | 0.54 | 0.70  (0.42, 1.17) | 0.17 | 0.69  (0.41, 1.16) | 0.16 |
| University undergraduate level qualification | - | - | 0.65  (0.41, 1.02) | 0.06 | 0.66  (0.41, 1.05) | 0.08 | 0.65  (0.40, 1.04) | 0.07 |
| University post-graduate level qualification | - | - | 0.45  (0.29, 0.70) | **<0.01** | 0.44  (0.28, 0.70) | **<0.01** | 0.45  (0.28, 0.71) | **<0.01** |
| Trauma (6-10) | - | - | - | - | 3.99  (1.47, 14.02) | **0.02** | 3.74  (1.38, 13.17) | **0.02** |
| Trauma (11-15) | - | - | - | - | 6.27  (2.35, 21.84) | **<0.01** | 5.56  (2.07, 19.46) | **<0.01** |
| Trauma (16-53) | - | - | - | - | 14.40  (5.50, 49.67) | **<0.01** | 11.60  (4.35, 40.47) | **<0.01** |
| 2+ MH/ND conditions^e^ | - | - | - | - | - | - | 1.54  (1.08, 2.20) | **0.02** |

Note. p-values for the following variables were adjusted for multiple testing correction: autistic traits, trauma, 2+MH/ND conditions. Values in bold indicate p-values less than 0.05.

^a^Model 1 = unadjusted. ^b^Model 2 = autistic traits and demographics. ^c^Model 3 = autistic traits demographics, and traumatic experiences. ^d^Model 4 = autistic traits, demographics, traumatic experiences, and two or more co-occurring mental health and/or neurodevelopmental conditions.^e^Mental health and/or neurodevelopmental conditions include: Anxiety disorder, Language delay, Attentional deficit hyperactivity disorder (ADHD)/Attentional deficit disorder (ADD), Obsessive compulsive disorder (OCD), Oppositional defiant disorder (ODD), Bipolar disorder, Panic disorder, Conduct disorder, Personality disorder, Depression, Post­traumatic stress disorder, Dyslexia, Schizophrenia/Psychosis, Dyspraxia/Developmental coordination disorder, Sensory processing disorder, Eating disorder, Social phobia/Social anxiety disorder, Intellectual disability, Specific phobia, Generalised anxiety disorder, Tourette syndrome/Tic disorder.

References

1. Griffiths S, Allison C, Kenny R, Holt R, Smith P, Baron-Cohen S. The Vulnerability Experiences Quotient (VEQ): A Study of Vulnerability, Mental Health and Life Satisfaction in Autistic Adults. Autism Res. 2019;12(10):1516–28.

2. Baron-Cohen S, Wheelwright S, Skinner R, Martin J, Clubley E. The Autism-Spectrum Quotient (AQ): Evidence from Asperger Syndrome/High-Functioning Autism, Males and Females, Scientists and Mathematicians. J Autism Dev Disord. 2001 Feb 1;31(1):5–17.
